# Herpetic anterior uveitis following Pfizer–BioNTech coronavirus disease 2019 vaccine: two case reports

**DOI:** 10.1186/s13256-022-03350-6

**Published:** 2022-03-25

**Authors:** José Manuel Ortiz-Egea, Cristina Gómez Sánchez, Andrés López-Jiménez, Olga Diego Navarro

**Affiliations:** 1 Ophthalmology Department, Complejo Hospitalario Universitario de Albacete, Albacete, Spain; 2 Ophthalmology Department, Hospital General Universitario Reina Sofía de Murcia, Murcia, Spain

**Keywords:** SARS-CoV2, COVID-19 vaccines, Herpes zoster, Uveitis anterior, Inflammation

## Abstract

**Purpose:**

To describe two cases of herpetic anterior uveitis after inoculation of the first dose of Pfizer–BioNTech coronavirus disease 2019 vaccine.

**Methods:**

Case 1: a healthy 92-year-old Caucasian woman developed symptomatic unilateral anterior uveitis for 3 days after Pfizer–BioNTech coronavirus disease 2019 vaccination (Pfizer Inc.). The episode fully resolved with topical and oral antiviral treatment. Case 2: a previously healthy 85-year-old Caucasian woman with left hemicranial signs of herpes zoster infection, associated with herpetic keratouveitis for 3 days after Pfizer–BioNTech coronavirus disease 2019 vaccination. Treatment with topical antibiotics and both oral and topical antiherpetic medication was administered, and she recovered successfully in 5 weeks.

**Conclusion:**

Clinicians should be aware of the possibility of eye inflammation in the form of herpetic reactivation after coronavirus disease 2019 vaccination.

## Background

On 11 December 2020, the Food and Drug Administration issued an emergency use authorization for the Pfizer–BioNTech vaccine to prevent COVID-19, administered as two doses separated by 21 days.

The Pfizer–BioNTech COVID-19 vaccine has been recommended to people 16 years of age or older, with a dose of 30 μg (0.3 ml), given twice, 21 days apart. Immunogenicity is provided for at least 119 days after the first vaccination and is 95% effective in preventing severe acute respiratory syndrome due to coronavirus 2 (SARS-CoV2) infection.

At this moment, millions of people have been and are being vaccinated with the Pfizer–BioNTech COVID-19 vaccine worldwide, and there are already publications describing isolated cases [1, 2] and others describing the development of uveitis and other ocular complications following administration of the COVID-19 vaccine [3]. The facts that T cells play an essential role in developing the specific humoral response of the vaccine [4] and that their depletion is also related to reactivation of latent herpesviruses [5] may represent the connection point between this inflammatory activation, which has resulted in uveitis, and the vaccine [4–6]. Therefore, the uveitis may have been a secondary and undesired effect of the Pfizer–BioNTech COVID-19 vaccine.

We present two cases of herpetic anterior keratouveitis within the first 72 hours after administration of the first dose of the Pfizer vaccine.

## Case reports

### Case 1

A 92-year-old Caucasian woman presented with ocular pain and redness in the left eye (LE) of 2 days evolution. Three days before the start of symptoms, she had received the first dose of the vaccine, denying systemic adverse reactions after the injection.

**Fig. 1:**
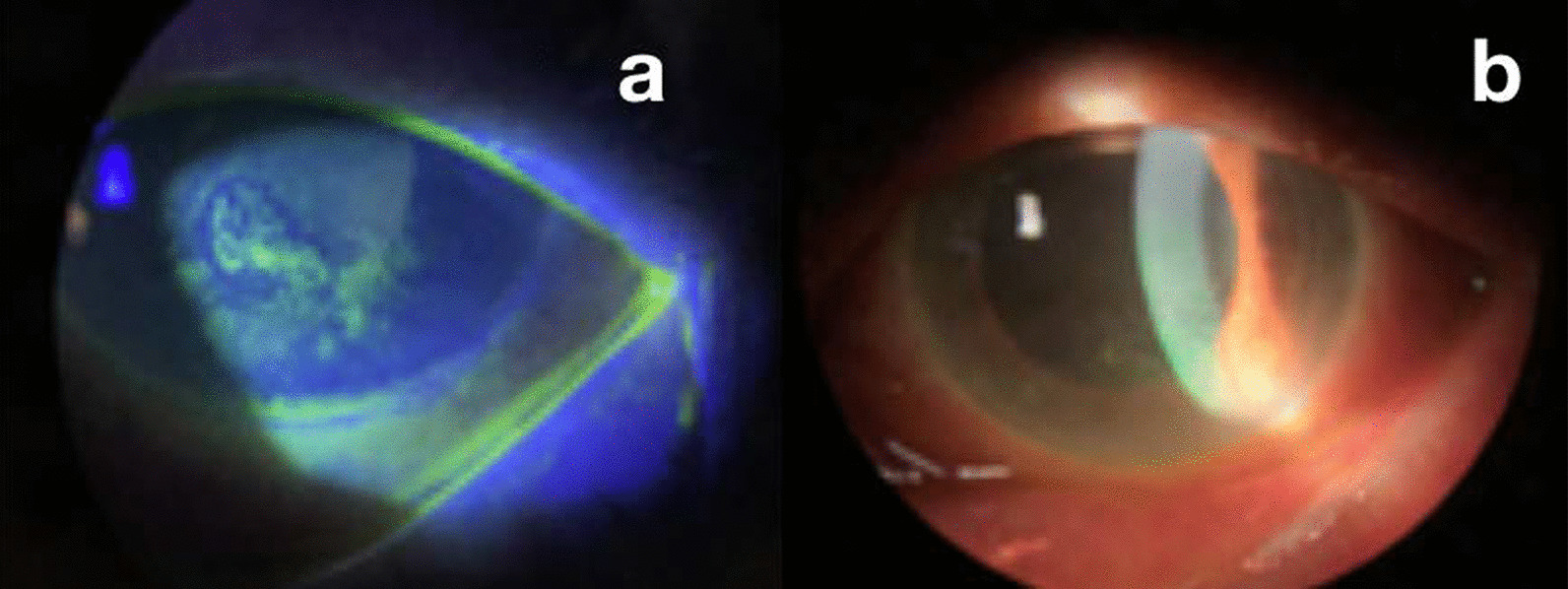
**a** Central and inferior positive fluorescein keratitis of dendritic morphology in the lower half of the cornea. **b** Image of the anterior ocular pole with corneal edema and mature brunescent cataract

Her best corrected visual acuity (BCVA) was counting fingers at 2 m (previously known BCVA of 1/10 in LE) and 6/10 in right eye (RE). The patient suffered from exudative age-associated macular degeneration in LE, which had been treated with intravitreal bevacizumab four months before. Slit-lamp examination showed mixed mild conjunctival hyperemia, intense corneal edema, with pigmented keratic precipitate (PKS) centrally and inferiorly, dendritic keratitis (Fig 1a), flare of 2/4+, and a mature brunescent cataract (Fig. 1b). No hypopyon or synechiae were seen. Intraocular pressure (IOP) was 17 mmHg in LE and 14 mmHg in RE. Antiviral treatment was prescribed: topical acyclovir five times daily, oral valaciclovir 500 mg every 24 hours (ClCr < 30 ml/minute), cycloplegic every 8 hours, and moxifloxacin eye drops every 4 hours. The patient experienced remarkable improvement in 4 weeks.

### Case 2

**Fig. 2:**
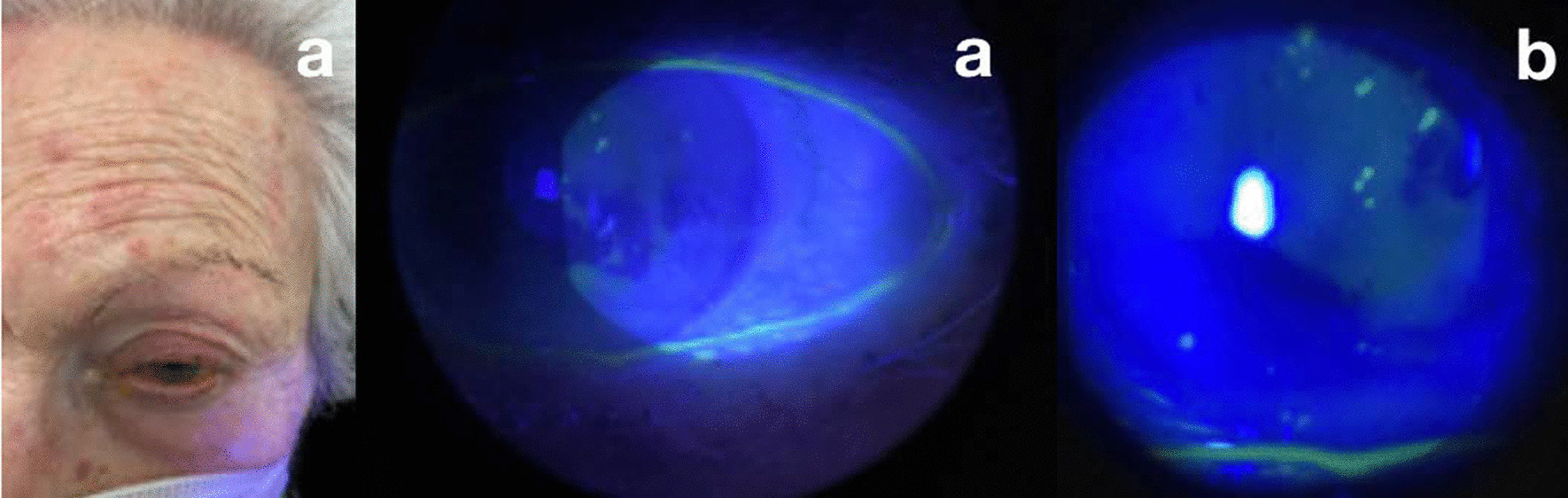
**a** Erythematous lesions in the left ophthalmic branch of the V cranial nerve associated with vesicles. **b** and **c** Mild conjunctival hyperemia and, in cornea, scattered epithelial lesions of small size with vesicular borders and fluorescein positivity

A 85-year-old Caucasian woman came to the emergency room alleging intense left hemicranial headache associated with ocular pain in her LE. She presented several erythematous lesions associated with vesicles in the area supplied by the left superior ophthalmic branch of the V cranial nerve. Her BCVA was 3/10 n LE and 7/10 in RE. Biomicroscopic evaluation of the LE showed mild conjunctival hyperemia and, in cornea, scattered epithelial lesions of small size with vesicular borders, fluorescein positivity (Fig. 2b, c), and flare of +/−. She was diagnosed with herpes zoster infection and treated with acyclovir ointment 5 times a day, 125 mg oral brivudine every 24 hours for 7 days, and moxifloxacin drops every 3 hours. Like in the first case, Pfizer COVID-19 vaccine had been administered in the previous 72 hours. No other signs or symptoms that could be related to the vaccine, or others of any kind, were observed.

## Discussion and conclusions

The most complete review of vaccine-associated uveitis was carried out by Benage and Fraunfelder in 2016, identifying 289 cases through bibliographic searches in Medline. One of the analyzed reviews found a high incidence of uveitis processes after vaccination [6]. Vaccination against hepatitis B virus (HBV), alone or in combination, was the most frequent (40.5%), followed by vaccination against human papilloma virus (15.6%), influenza virus (9.7%), Bacille–Calmette–Guérin (7.3%), measles–mumps–rubella alone or in combination (4.8%), chickenpox alone or in combination (4.8%), and hepatitis A virus alone or in combination (2.4%). Among the 276 cases in which the gender of the patients was reported, 72.1% were women, the mean age at onset was 30 years (range 2 months to 86 years), and the median duration between vaccination and the onset of uveitis was 16 days (range: 1 day to 6 years; SD = 362 days). Studying the cases in which treatment and response were provided, the most common uveitis was anterior and responded satisfactorily to topical steroid treatment, like in our two cases.

COVID-19 vaccines can cause mild adverse effects after the first or second dose, including pain, redness, or swelling at the injection site, fever, fatigue, headache, muscle pain, nausea, vomiting, itching, chills, joint pain, and in rare cases, anaphylactic shock [7]. The occurrence of adverse effects is reported to be less for the Pfizer–BioNTech vaccine compared with the Moderna vaccine [3, 8].

As we know, vaccines and their adjuvants are specifically designed to interact with the immune system and activate it, but the precise pathogenesis of vaccine-associated uveitis remains unclear. Proposed mechanisms have included molecular mimicry and antigen-specific cell and antibody-mediated hypersensitivity reactions [9]. However, these mechanisms are not limited to vaccines. It has been suggested that vaccines share nonspecific mechanisms with postinfectious and drug-induced uveitis in their pathogenesis. Two probable specific mechanisms of vaccines have been described and seem to be of special relevance to uveitic activation [9]. The first mechanism, applicable to live attenuated vaccines, implies a possible direct infection by the attenuated but still active virus strain. Although rare, such infections have been documented, even in the context of uveitis [10]. The second mechanism involves inflammation induced by one or more adjuvant (typically aluminum salts) commonly used in inactivated or subunit/conjugate vaccines, containing only components or parts of the target pathogen [11].

The Pfizer–BioNTech COVID-19 vaccine triggers a CD8 T-cell immune response [4]. This cellular response could provide additional protection against SARS-CoV2 infection, but at the same time, these activated T cells could trigger other immune disorders in other parts of the body. How these lymphocytes activated by the Pfizer–BioNTech COVID-19 vaccine can reach ocular structures is explained by experimental models of autoimmune uveitis in Lewis rats [12]. To control the migration of autoreactive T lymphocytes to different organs, control T lymphocytes were injected specific to R14 (amino acids 1169-1191 of antigen-activated retinoid-binding protein interphotoreceptors) intravenously or ovalbumin (OVA) with intravenously transduced green fluorescent protein (GFP) in naive rats. Migration of fluorescent T cells was observed by daily intravital fluorescence microscopy of the iris [13]. The first GFP+ T cells, regardless of their specificity for autoantigen or OVA, were extravasated and entered the iris tissue within 30 minutes after injection. The number of GFP+ cells of any specificity increased gradually during the first 3 days. They describe that, at 3 days, the amount of OVA-specific GFP+ T cells decreased, while R14-specific T cells increased, accompanied by infiltration of inflammatory GFP cells. It is at this time that the clinical disease begins, with ophthalmoscopically visible infiltration of leukocytes.

At the same time, we know the complexity of controlling herpesvirus latency in neurons, in which innate intranodal immunity (satellite glial cells that interact with neurons) and adaptive immunity (T cells) are essential. While innate immunity is more likely to be sufficient to prevent varicela zoster virus from escaping dormancy, herpes simplex virus-1 (HSV-1)-specific CD8 T cells are of additional importance in preventing HSV-1 reactivation [5]. Although there are several factors that determine the incidence and spectrum of ocular herpetic diseases, such as immune status, age, genetic predisposition, time and route of infection, and potentially virus strain, we also know that the Pfizer–BioNTech COVID-19 vaccine can generate immune activation of T cells (CD8) [4] that 3 days later [12] can reach the ocular structures and generate ocular inflammation.

Vaccine-induced immunomodulation has been observed [4–6]. There are published cases that provide evidence of herpesvirus reactivation infections after vaccines against other (nonherpetic) viruses [14, 15]. In herpes viruses, viral replication and host morbidity are limited by pathogenic and host factors, allowing the virus to persist throughout the life of the host. Numerous host defense mechanisms work to limit herpetic infection. The host detects DNA, RNA, and viral proteins through pathogen recognition receptors, such as toll-like receptors. Activation of these receptors results in the release of proinflammatory cytokines, particularly type II interferons and IL-1b, which inhibit viral replication, promote the destruction of infected cells, and recruit phagocytes to kill infected cells [15].

Resident microglia and infiltrating CD8+ T cells provide additional defense in the form of direct cellular toxicity and antiviral cytokines. Recurrent subclinical viral replication elicits a local inflammatory response that triggers immune surveillance of infected trigeminal ganglia [14]. This phenomenon, together with viral regulatory elements, is essential to suppress a clinically apparent infection. Thus, clinical disease represents a tipping of this balance in favor of viral proliferation.

The proposed immunological mechanisms for why SARS-COV-2 vaccination can trigger herpetic reactivation include molecular mimicry, whereby immunity to a vaccine peptide triggers an immune response against a similar host protein, and autoinflammation. Furthermore, the local immune response at the vaccination site and subsequent systemic activation could distract attention from normal immune surveillance, so that the number of T cells normally present near infected neurons is effectively reduced, resulting in loss of immune control [14].

In our patients, there was a reproducible temporal relationship between vaccination against SARS-CoV-2 and herpetic reactivation. The exact mechanism remains elusive, but vaccine-induced immunomodulation may be involved. Published literature [1–3, 6, 14, 15] and our cases together with the unknown frequency of uveitis after vaccination against SARS-COV-2 reveal not the dangers of vaccination against SARS-COV-2 but the complexity of the interface between herpes virus and the host, calling for further study and a search for a causal relationship.

Vaccination with COVID-19 may be followed by reactivation of herpetic keratitis in patients with previous herpetic keratitis or keratouveitis. Changes in immune status, including lymphocyte depletion, may lead to herpes reactivation [12–15]. Therefore, prophylactic antiviral treatment with oral valacyclovir could be considered, at least for high-risk patients with several previous episodes of herpetic uveitis.

Epidemiological and biological studies are needed to elucidate the possible link between vaccination and reactivation of herpesvirus infections. Clinicians should be aware of the possibility of eye inflammation in the form of herpetic reactivation after COVID-19 vaccination.

## Data Availability

Not applicable.

## Abbreviations

BCVA: Best corrected visual acuity
COVID-19: Coronavirus disease 2019
GFP: Green fluorescent protein
HSV-1: Herpes simplex virus-1
LE: Left eye
OVA: Ovalbumin
RE: Right eye
SARS-CoV2: Severe acute respiratory syndrome coronavirus 2
